# Giant Cell Myocarditis: Not Always a Presentation of Cardiogenic Shock

**DOI:** 10.1155/2015/173826

**Published:** 2015-07-16

**Authors:** Rose Tompkins, William J. Cole, Barry P. Rosenzweig, Leon Axel, Sripal Bangalore, Anuradha Lala

**Affiliations:** ^1^Department of Cardiology, New York University Langone Medical Center, New York, NY 10016, USA; ^2^Department of Radiology, New York University School of Medicine, New York, NY 10016, USA

## Abstract

Giant cell myocarditis is a rare and often fatal disease. The most obvious presentation often described in the literature is one of rapid hemodynamic deterioration due to cardiogenic shock necessitating urgent consideration of mechanical circulatory support and heart transplantation. We present the case of a 60-year-old man whose initial presentation was consistent with myopericarditis but who went on to develop a rapid decline in left ventricular systolic function without overt hemodynamic compromise or dramatic symptomatology. Giant cell myocarditis was confirmed via endomyocardial biopsy. Combined immunosuppression with corticosteroids and calcineurin inhibitor resulted in resolution of symptoms and sustained recovery of left ventricular function one year later. Our case highlights that giant cell myocarditis does not always present with cardiogenic shock and should be considered in the evaluation of new onset cardiomyopathy of uncertain etiology as a timely diagnosis has distinct clinical implications on management and prognosis.

## 1. Introduction

Giant cell myocarditis (GCM) is a rare and often fatal disease with the most obvious presentation being a rapid hemodynamic deterioration with declining left ventricular (LV) systolic function and cardiogenic shock [1]. We report the case of a patient with confirmed GCM who did not present in fulminant heart failure highlighting the variability of presentation and potential for underrecognition of GCM, which could greatly impact subsequent treatment and prognosis.

## 2. Case Presentation

**Figure 1:**
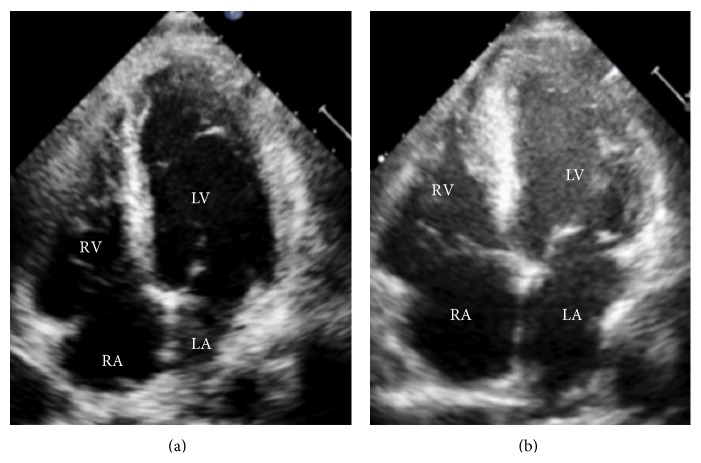
Transthoracic echocardiogram (apical four-chamber view). (a) Hospital day 1: normal ventricular function, normal wall thickness. (b) Hospital day 7: left ventricular systolic function is severely reduced, wall thickness is increased, and echocontrast is present in the left ventricle consistent with stasis of blood flow. LV: left ventricle, RV: right ventricle, LA: left atrium, and RA: right atrium.

**Figure 2:**
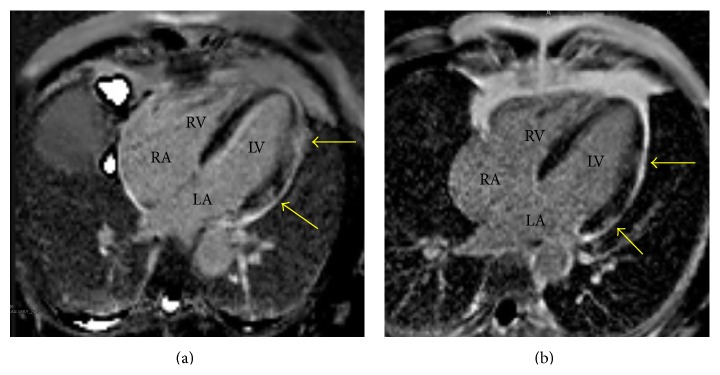
Cardiac magnetic resonance image (four-chamber view). (a) Presentation: patchy areas of late gadolinium enhancement (LGE) were detected transmurally in the mid lateral left ventricular (LV) wall and in the midwall with extension to the subepicardial in lateral and anterior LV walls near the base consistent with diffuse inflammation in a noncoronary distribution (arrows). (b) 6 weeks after presentation: markedly reduced LGE (arrows) consistent with improvement in inflammation without significant residual fibrosis. LV: left ventricle, RV: right ventricle, LA: left atrium, and RA: right atrium.

A 60-year-old previously healthy African American man complained of progressively worsening chest pain for five days. He had no known cardiovascular risk factors. His physical examination was normal. Electrocardiogram showed diffuse ST elevations with associated cardiac troponin I of 7.6 ng/mL (reference range ≤ 0.04 ng/mL). Emergent cardiac catheterization revealed angiographically normal coronary arteries. On hospital day one, a transthoracic echocardiogram (TTE) was notable for normal left ventricular function, chamber size, and wall thickness (Figure 1(a); see Movie 1 in Supplementary Material available online at http://dx.doi.org/10.1155/2015/173826). This was followed by a contrast-enhanced cardiac magnetic resonance (CMR) imaging that showed normal left ventricular (LV) systolic function with an ejection fraction (EF) of 55% and multiple patchy areas of transmural and midwall late gadolinium enhancement (LGE) in a noncoronary distribution (Figure 2(a)). He was treated for presumed myopericarditis with nonsteroidal anti-inflammatory drugs (NSAIDs). His symptoms resolved and his troponin levels decreased.

On hospital day five, the patient complained of new dyspnea on exertion, paroxysmal nocturnal dyspnea, and recurrent chest discomfort. His blood pressure decreased from 130/80 on admission to 100/70 mmHg. His rhythm was sinus tachycardia with a heart rate (HR) of 110 bpm. The jugular venous pressure was 7 cm H_2_O and hepatojugular reflux was evidenced. There was no gallop sound or murmur, the lungs were clear, and his extremities were warm without peripheral edema. TTE now showed a mildly reduced EF of 45% with hypokinesis of the left ventricular apical anterior and apical lateral walls. Notably, the left ventricular wall motion abnormalities were in the areas of myocardium with LGE seen on CMR imaging. Low dose furosemide was initiated with improvement in his symptoms.

**Figure 3:**
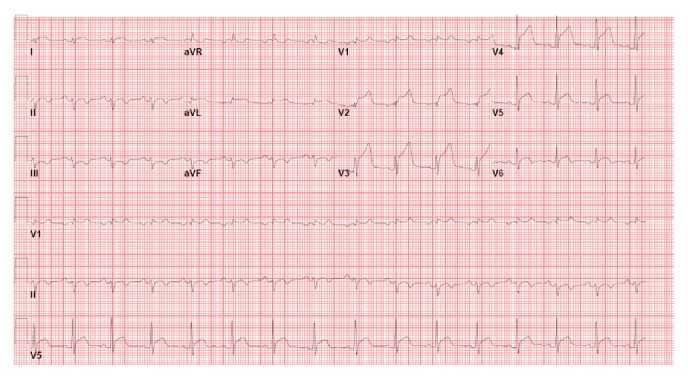
Twelve-lead electrocardiogram on hospital day 7 showed persistent diffuse ST elevation.

Over the next two days, serial TTEs showed a rapid decline in LV systolic function from 45% to 25%, increased wall thickness suggestive of myocardial edema, and spontaneous echo contrast in the left ventricle consistent with stasis of blood flow (Figure 1(b), Movie 2). Concomitantly, troponin levels rose again to a peak of 9.6 ng/mL associated with persistence of diffuse ST elevations on electrocardiogram (Figure 3). Inflammatory markers, including erythrocyte sedimentation rate (ESR) and C-reactive protein (CRP), were elevated at 135 mm/hr and 329 mg/L, respectively. Renal function and lactate levels remained within normal limits. No significant arrhythmia or ventricular ectopy was noted. His mild symptoms were controlled with low dose oral furosemide alone.

**Figure 4:**
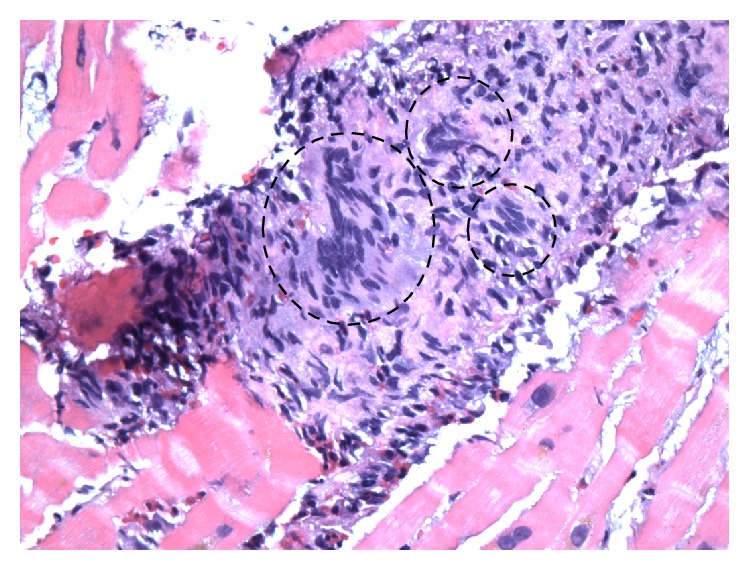
Microscopic examination from the endomyocardial biopsy of the right ventricle showing myocardial necrosis with inflammatory infiltrate containing multinucleated giant cells (within circled areas) (H&E, orig. ×40).

Given the rapid and dramatic decline in LV systolic function, an endomyocardial biopsy was performed. Microscopic analysis revealed widespread necrosis and an inflammatory infiltrate comprising neutrophils, eosinophils, and multinucleated giant cells consistent with the pathological diagnosis of giant cell myocarditis (GCM) (Figure 4). Immunosuppression was initiated with intravenous methylprednisolone for three days followed by a slow oral prednisone taper. Cyclosporine was added in conjunction with low dose ACE inhibition and diuretic therapy.

Two weeks following hospital discharge, the patient was free of heart failure (HF) symptoms. TTE showed improved contractility with an EF of 45%. Repeat CMR 6 weeks later demonstrated residual patchy areas of midwall late gadolinium enhancement but the overall extent of enhancement was significantly reduced. CMR-measured EF had normalized to 55% with resolution of all wall motion abnormalities and no evidence of diastolic dysfunction (Figure 2(b)). The patient underwent stress testing with echocardiographic imaging five months after initial presentation, during which he completed 12 minutes (12.4 METS) of a Bruce protocol without exertional symptoms or ventricular ectopy. Echocardiographic imaging demonstrated an appropriate increase in myocardial contractility with exercise. Currently, nearly 12 months after his initial presentation, he remains asymptomatic on guideline-directed medical therapy (GDMT) for HF and combination immunosuppression with cyclosporine (goal trough level 100–120 ng/mL) and prednisone at a maintenance dose of 5 mg daily. If symptoms recur, then repeat imaging with another endomyocardial biopsy may be required.

## 3. Discussion

Idiopathic GCM is a rare and often fatal disease [1]. Initial presentation can be one of rapidly progressive HF, ventricular arrhythmia, heart block, and/or symptoms mimicking acute coronary syndrome as seen in this case presentation [1, 2]. GCM is characterized histopathologically as a diffuse or multifocal inflammatory infiltrate with multinucleated giant cells associated with myocardial necrosis and an absence of sarcoid-like granulomas [1, 3]. Pathology remains the cornerstone of diagnosis [3]. Once the diagnosis is confirmed, there is considerable evidence to support the use of combined immunosuppression with calcineurin inhibition and corticosteroid therapy, as opposed to corticosteroids alone, in order to prolong transplant-free survival [1–4].

A rapid hemodynamic deterioration with declining LV systolic function and cardiogenic shock is the most obvious presentation of GCM requiring urgent consideration of inotropes, mechanical circulatory support, and transplant, in addition to immunotherapy [5–7]. The current report highlights the variability seen in the presentation of GCM. Our patient's initial presentation was not consistent with fulminant myocarditis, and although there was a rapid and severe decline in LV systolic function, he remained only mildly symptomatic with minimal signs of hemodynamic compromise. Such presentations can be misleading and may contribute to the underrecognition of GCM. Selected reports of GCM describe only mildly reduced LV systolic function in some patients while others had multiple admissions for HF prior to subsequent rapid ventricular deterioration [2, 5, 8, 9]. Cooper Jr. et al. reported that more than 50% of their GCM cohort had an EF > 45% at the time of diagnosis [9]. In addition, Kandolin et al. showed that 26% of their registry patients with confirmed GCM had an EF ≥ 50% [2].

A strong index of suspicion for GCM is required in the appropriate clinical context with less fulminant presentations, since the diagnosis has distinct implications for treatment and prognosis [3]. As shown in the Multicenter Giant Cell Myocarditis Registry, transplant-free survival is dismal without combined immunosuppression (1.8 months versus 33.5 months, *P* < .001) [1]. Although patients with preserved systolic function may have improved transplant-free survival compared to those patients with reduced LV function, relapse rates are high and recurrence has been described with discontinuation of immunotherapy up to 8 years following the initial diagnosis [2, 4, 9]. Current data support treatment with the combination of calcineurin inhibitors and corticosteroids, regardless of ventricular function [1–4]. Optimal treatment duration, however, remains undefined. Chronic immunosuppression is not without risks; it is associated with major adverse events and requires routine monitoring of renal function, bone density, prophylaxis for infection, and surveillance for neoplastic disease for those who survive in the long term.

## 4. Conclusion

GCM does not always present with rapid hemodynamic deterioration and cardiogenic shock but can also be diagnosed in patients with initially normal left ventricular function and among those with nonfulminant acute HF of uncertain etiology. Establishing the diagnosis of GCM is critical for the management and prognosis of the disease as combination immunosuppression versus corticosteroids alone significantly improves transplant-free survival [1–4, 9]. Current data suggest that GCM may be a life-long, chronic disease [2, 4]. Recommendations for long-term immunosuppression in asymptomatic patients who do not undergo cardiac transplantation remain undefined. Therefore, the risks and benefits of long-term immunotherapy should be considered and management decisions individualized.

## Supplementary Material

Movie 1: Transthoracic echocardiogram (TTE) in the apical four chamber view from hospital day 1 shows normal ventricular function, normal ventricular size, and normal ventricular wall thickness.
Movie 2: Repeat transthoracic echocardiogram (TTE) in the apical four chamber view from hospital day 7 shows a dramatic decline in left ventricular systolic function with a severely depressed EF 25%, thickened ventricular walls consistent with edema, and dilation and hypokinesis of the right ventricle. Spontaneous echocontrast or "smoke" can be seen in the left ventricle due to low flow and stasis.
